# Traffic Noise Annoyance in the Population of North Mexico: Case Study on the Daytime Period in the City of Matamoros

**DOI:** 10.3389/fpsyg.2021.657428

**Published:** 2021-05-24

**Authors:** Benito Zamorano-González, Fabiola Pena-Cardenas, Yolanda Velázquez-Narváez, Víctor Parra-Sierra, José Ignacio Vargas-Martínez, Oscar Monreal-Aranda, Lucía Ruíz-Ramos

**Affiliations:** Unidad Académica Multidisciplinaria Matamoros, Universidad Autónoma de Tamaulipas, Matamoros, Mexico

**Keywords:** traffic noise, annoyance noise, perception, noise, urban soundscape

## Abstract

**Aim:** The presence of noise in urban environments is rarely considered a factor that causes damage to the environment. The primary generating source is transportation means, with vehicles being the ones that affect cities the most. Traffic noise has a particular influence on the quality of life of those who are exposed to it and can cause health alterations ranging from annoyance to cardiovascular diseases. This study aims to describe the relationship between the traffic noise level and the perceived annoyance in the inhabitants of a city on the Northern Border of Mexico. The work carried out in a city represents the vulnerability characteristics: economic, social, and migratory of its sizable portion of the inhabitants. Due to that, it is impossible to identify precisely the number of residents as the number of vehicles in circulation.

**Methods:** The streets and avenues with an annual average daily traffic of more than 1,000 vehicles were considered for the measurement of traffic noise. The equipment used was a vehicle gauge with non-invasive speed radar; type I integrating sound level meters, with their respective gauges and tripods. A questionnaire was applied to people living within 250 m of the streets and avenues in which the noise was measured.

**Results:** The noise measurement found a parameter of LA_eq_ estimated for 12 h during the day, exceeding 70 dBA. The data received from the questionnaire were statistically tested by using Pearson's correlation tests. A total number of 2,350 people were participated, of whom 1,378 were women (58.6%) and 972 were men (41.4%). The age of participants is ranged from 18 to 75 years. The overall perception of traffic noise annoyance identified that 1,131 participants (48.1%) responded “Yes” as they considered the noise annoying. Participants who responded “No” as well as those who responded “Do not know” resulted in a total of 1,219 people (51.9%).

**Conclusion:** The results show that the population is desensitized to traffic noise and does not perceive it as an annoyance. The flow of vehicles and the type of vehicles are the significant factors for the propagation and increase in the traffic noise levels. Women present a considerable appreciation of traffic noise perception instead of younger people who demonstrate a higher tolerance to high-level exposure. This reflects the lack of information of the population around the noise problem and its effects.

## Introduction

Technological advances, industry, and everyday activities influence noise in the urban regions. In addition, transport, construction sites, and rapid population growth are responsible for generating acoustic variations in urban centers. This means that all these activities break the natural balance of the environment, causing damage to any individual with a particular time and exposure level. The environmental noise is currently one of the main types of pollution in large cities, regardless of the development level (Echeverry Velasquez, 2011; Cohen and Castillo, 2017). Therefore, it is essential to recognize that as the population is concentrated in a particular area, in addition to the type of activities they carry out, the presence of environmental noise also increases (Zamorano González et al., 2015).

The population faces an environmental problem that is rarely conscious. Unlike other environmental pollution types, it is characterized by its progressiveness and the generation of adverse, direct, and cumulative effects on health. Daily exposure to loud sounds, even those of short duration, causes damage to the auditory threshold of humans, which increases with age (Alvarado et al., 2019). Some studies describe some health alterations caused by noise, which aim to explain the consequences of exposure to noise for a long time, particularly noise generated through transport, such as trains, planes, cars, and motorcycles (Vienneau et al., 2015; Christensen et al., 2017; Mueller et al., 2017; Park and Lee, 2017; Oh et al., 2019). Other proposals explain that transport noise can influence intellectual activities, disrupting reading, comprehension, and even memorization (Halin, 2016).

The literature review describes the need to identify health hazards and relate them to a hearing problem. Even the World Health Organization highlights the influence that vehicular traffic noise has on irritability, interference in communication, the sensation of annoyance. It can even affect the work performance due to fatigue caused by disrupting sleep. Insight of these consequences, noise is an environmental problem that is generally detrimental to the lifestyle and quality of life (Fyhri and Aasvang, 2010; World Health Organization, 2011; Zamorano González et al., 2015; Guski et al., 2016; Gasco et al., 2020). In this way, other studies agree that noise affects the quality of life, well-being, and mental health but require the inclusion of other transport means, as well as a greater methodological depth to improve their findings (Clark and Paunovic, 2018).

In the context of Latin America, the work of some authors stands out. In Brazil, studies on acoustic comfort and quality of life have been carried out in the following areas (Levandoski and Trombetta Zannin, 2020): noise annoyance perception (Paiva et al., 2019), the development of noise maps (Kirrian Fiedler and Trombetta Zannin, 2015), design of prediction models (Oliveira do Nascimento et al., 2021), and the noise generated by different means of transportation (Bunn and Trombetta Zannin, 2016). In Chile, studies focus on the elaboration of noise maps (Suárez and Barros, 2014), noise mapping methods (Bastián-Monarca et al., 2016), road noise estimation (Rey Gozalo et al., 2020), and the design of smartphone applications for noise monitoring (Aumond et al., 2020).

In case of Mexico, the work reported in English is minimal. For example, the study describes the noise level above 96 dBA in commercial areas (Environmental Noise in Mexico City, 2019). Another study defines the environmental noise as annoying but included it in a set of environmental aggressors without performing the measurements (Sánchez-Arias et al., 2019). While there are other studies, its diffusion in Spanish limits its dissemination at the international plane. Some examples are the qualitative study of different noise types exceeding 70 dBA (Rodríguez-Manzo and Juárez González, 2020), noise pollution generated in a border town center (Zamorano González et al., 2015), as well as noise associated with sleep quality and performance (Zamorano González et al., 2019). The previously mentioned evidence is the need for studies on noise, especially the noise generated by traffic and the impacts it has on the population, which are the results of the limited effort by a few authors in a country such as Mexico with a large population and geographical dimensions.

The regulatory framework in Mexico attempts to reduce the noise generation; however, the laws presented are particular to a specific sector, as is the instance of the standard that regulates noise inside workplaces (NOM-011-STPS-2001, 2001). Mexican Official Norm 081 defines the study and control of noise from fixed sources in the environmental aspect, published in 1995. According to the type of noise source, they only update the table of maximum noise exposure levels over the years (NOM 081 SEMARNAT, 1994, 2013). Other types of noise standards evaluate the levels generated by the automobile vehicles. However, their measurement must be performed in the verification centers, leaving aside the soundscape when the vehicles are in circulation.

In addition to the normative limitations, the social and cultural aspects of the populations located in border cities present a cultural diversity, a consequence of the migratory movements between the United States and Latin America. In this sense, the towns give a gap of inequalities, both in social rights and economic development, becoming vulnerable areas due to their lower level of well-being and quality of life (Consejo Nacional de Evaluación de la Política de Desarrollo Social, 2018).

Therefore, it is common for people to express distrust toward the institutions derived from ethnic and linguistic differences, representing an attitudinal barrier on the part of the participants (Calva Sánchez and Alarcón Acosta, 2018). This shows that the cultural differences can influence when carrying out any intervention or studies that address the environmental landscape (Zijlema et al., 2020).

The importance of carrying on the environmental noise studies concerning social aspects allows determining the quality of life of the inhabitants in a particular area (Paiva et al., 2019). The perception of noise nuisance of the population can be altered by personal variables, such as health status, age, and gender. Nevertheless, certain external conditions also influence the number of neighbors and the type of housing construction. For this reason, the population should be aware of being able to determine the objectives and subjective variables that influence the presence of noise, but likewise of its consequences (Koprowska et al., 2018).

The present study intended to cover a part of the research gap in Mexico and to join the effort of other authors in Latin America to study the environmental noise problem. This main objective of this study is to describe the relationship between the traffic noise level and the perception of annoyance in the inhabitants of a city on the Northern Border of Mexico.

## Materials and Methods

The research was developed during the period April to September 2016, in H. Matamoros, Tamaulipas, located in the northeast of Mexico and has a territorial extension of 4045.62 km^2^. According to the data registered in the last available census, the city has 520,367 inhabitants, where 51.3% were women. The vehicle registry indicates a total of 132,938 cars (Instituto Nacional de Estadística y Geografía, 2015). These quantities could triple due to the migratory characteristics of the city; the first is due to the number of people who have the objective of crossing to the United States and do not achieve it, who remain indefinitely in the city. The second characteristic is a consequence of the deportation processes. A group of people establishes their residence “temporarily” with the firm desire to return to the United States; however, the wait could last for years. The third characteristic of importance of this study is the lack of control for foreign-origin vehicles since many cars cross the border but do not return to the United States (Figueroa-Hernández and Pérez-Soto, 2011).

At the time of this study, it was impossible to obtain the official records to allow random sampling. For this reason, an intentional procedure was followed for the selection of the assessment areas. The intersections with the highest traffic flow in the diverse sectors of the city were selected, considering the opinion of the research team. These streets possess the characteristics to be classified as secondary streets or roads because they are utilized for small length and with speeds in the range of 25–62 m/h. According to the law, it is essential to emphasize that this categorization refers to the antiquity of more than 15 years (Secretaría de Comunicaciones y Transportes, 2006).

The ideal scenario for vehicle capacity is to perform them continuously during a year, i.e., 24 h a day. However, it represents a significant consumption of resources, so the traffic samples are used as a strategy to optimize them. In this study, we installed a non-invasive speed camera, model SafePace, from the brand Traffic Logix, which records the data in the internal memory and allows it to be transferred to a computer via Bluetooth, with the software SP-Data. The measured vehicle capacity at each intersection was counted for 1 week. Initially, traffic dense identified 15 intersections, but the observation discarded four roads due to the maintenance and road repairs.

The present study used a reference for the measurement of traffic noise, the ISO-1996-1-2016 standard (International Standards Organization, 2016), in the absence of a national standard describing the environmental noise assessment procedures. It is worth mentioning that the standard points out the installation of type I sound level meters, which must be installed at the height of 5 m and 1½ m away from any facade. Besides, it describes the premise that noise levels may be evaluated at the height of 1½ m in open spaces, ensuring no noise reflecting barrier (Barrigón Morillas et al., 2016).

For this purpose, Quest 3M integrated the type I sound level meter with their respective calibrators. Due to the difficulty of locating the sound level meters at the height described previously, tripods of 4 m in height were used and located at a distance of 3 m from any facade or wall that could reflect the sound. In this case, the position will not represent a risk for the field personnel, and we tried to ensure that the measurements were as close as possible to those described by the standard.

Noise measurement is carried out in three periods of the day, from Monday to Friday: the first period is from 6.30 to 7.30 a.m.; the second is from 12.30 to 1.30 in the afternoon; and the third is from 5.30 to 6.30 p.m. The field measurements allowed to obtain the acoustic parameters as follows: equivalent continuous sound pressure level (LA_eq12_), the maximum sound level (LA_max_), and the minimum sound level (LA_min_), which were transferred directly from the sound level meter through an USB connection to a computer.

In addition, during the fieldwork, the research team verified that, in the noise evaluations, the weather conditions were free of rain and thunder; the device also used a portable anemometer to verify that the wind speed was <3 m/s. When the weather conditions were different, the measurement day was canceled and rescheduled.

**Figure 1:**
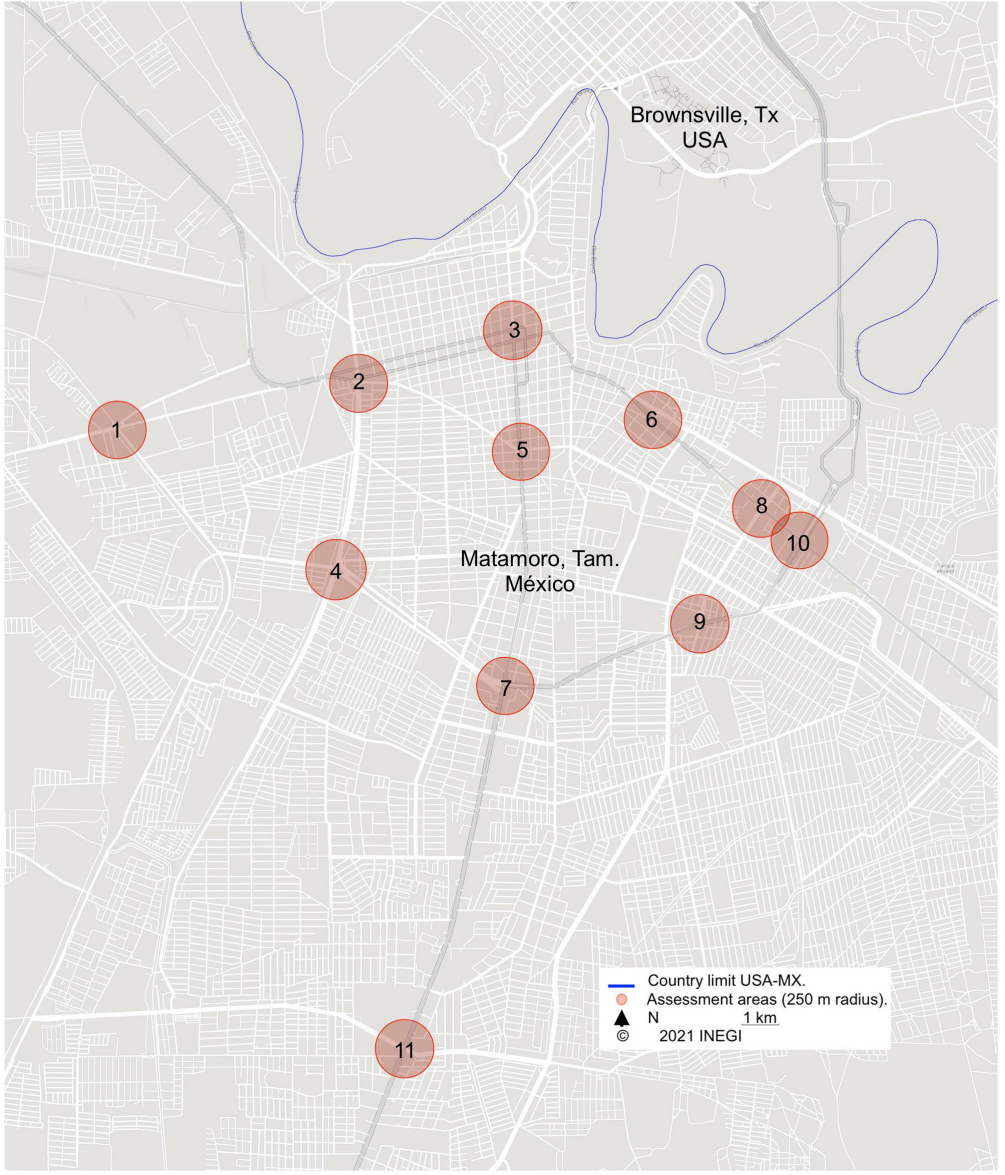
Assessment areas.

A questionnaire was used to obtain the information from the population about traffic noise perception, with seven questions used and requesting specific demographic information. The application of the questionnaire was carried out through the visits of the surveyors to each home within a radius of 250 m from the road intersection where the field personnel took the noise measurement. Figure 1 shows the distribution of the evaluation points of this study.

## Results

Table 1 represents the information obtained from the vehicle gauge. It is observed that the values calculated for the average weekly traffic (AWT) and the values of the annual average daily traffic (AADT) exceed 1,000 vehicles on the different roads. Similarly, the data obtained from the sound level meter are presented, where it is observed that, in all zones, the LA_eq_ parameters estimated for 12 h during the day exceed 70 dBA, as well as the minimum values fluctuate between 51.8 and 61.8 dBA, while the maximum values are between 86.7 and 103.9 dBA.

**Table 1:** Measurement results.

**Area**	**Vehicles type**		**Traffic volume**		**Noise parameters**		
	**Lightweight**	**Heavy**	**AWT**	**AADT**	**L_**Aeq**_**	**L_**AMin**_**	**L_**AMax**_**
1	1,545	178	1,723	1,617	72.3	51.9	97.8
2	2,375	170	2,545	2,305	74.7	60.4	98.2
3	1,246	42	1,288	1,175	70.5	51.8	94.9
4	1,904	187	2,091	1,935	74.5	58.7	98.5
5	2,437	47	2,484	2,228	74.7	60.4	98.2
6	1,461	25	1,486	1,394	71.9	56.8	103.9
7	2,164	258	2,422	2,210	75.6	58.3	97.9
8	1,885	48	1,933	1,793	71.7	58.6	86.7
9	1,408	47	1,455	1,344	73.2	61.8	99.1
10	2,168	150	2,318	2,025	72.1	59.7	94
11	1,832	361	2,193	2,059	74.8	60.4	95.2

*Extracted values of sound level meter and vehicular gauging device*.

As part of the fieldwork, we visited people living within the area to collect data. The questionnaire used requested the sociodemographic information such as gender, age, marital and school status, and work situation. Of note, 2,350 participants were interviewed in their homes, of which 1,378 were women (58.6%) and 972 were men (41.4%). The age of those with the highest participation was within a range of 18–30 years with a total of 867 participants (36.9%), while those over the age of 61 years were 252 participants (10.7%), being the range with the lowest participation. Due to many participants under the age of 30 years, the single marital status presented the involvement of 931 people (39.6%), unlike the widowed marital status, i.e., a total of 134 people (5.7%). The educational attainment level is represented by 561 people (23.9%) at the secondary level, while 58 people (2.5%) responded that they have some graduate degree. The employment situation of the participants found that 1,153 people (49.1%) have some employment, while only 7 people (0.3%) are unemployed or on strike. Table 2 represents a cross-tabulation between the demographic data and traffic noise.

**Table 2:** Demographic data and noise perception.

	**Noise perception**							
	**No**		**Yes**		**I don't**		**Total**	
**Gender**	**#**	**%**	**#**	**%**	**#**	**%**	**#**	**%**
Male	394	16.8	454	19.3	124	5.3	972	41.4
Female	518	22	677	28.8	183	7.8	1,378	58.6
Total	912	38.8	1,131	48.1	307	13.1	2,350	100
**Age**	**#**	**%**	**#**	**%**	**#**	**%**	**#**	**%**
18–30	376	16.0	361	15.4	130	5.5	867	36.9
31–45	252	10.7	300	12.8	79	3.4	631	26.9
46–60	201	8.6	326	13.9	73	3.1	600	25.5
>60	83	3.5	144	6.1	25	1.1	252	10.7
Total	912	38.8	1,131	48.1	307	13.1	2,350	100
**Education level**	**#**	**%**	**#**	**%**	**#**	**%**	**#**	**%**
Uneducated	95	4.0	215	9.1	55	2.3	365	15.5
Elementary	189	8.0	262	11.1	90	3.8	541	23.0
Junior High School	234	10.0	254	10.8	73	3.1	561	23.9
High School	210	8.9	193	8.2	57	2.4	460	19.6
University	172	7.3	169	7.2	24	1.0	365	15.5
Post-graduate	12	0.5	38	1.6	8	0.3	58	2.5
Total	912	38.8	1,131	48.1	307	13.1	2,350	100
**Employment status**	**#**	**%**	**#**	**%**	**#**	**%**	**#**	**%**
Unemployed	114	4.9	152	6.5	63	2.7	329	14.0
Worker	486	20.7	523	22.3	144	6.1	1,153	49.1
Strike	4	0.2	2	0.1	1	0.0	7	0.3
Retired	34	1.4	85	3.6	15	0.6	134	5.7
Housewife	189	8.0	282	12.0	59	2.5	530	22.6
Student	85	3.6	87	3.7	25	1.1	197	8.4
Total	912	38.8	1,131	48.1	307	13.1	2,350	100
**Marital status**	**#**	**%**	**#**	**%**	**#**	**%**	**#**	**%**
Single	314	13.4	471	20.0	146	6.2	931	39.6
Married	497	21.1	519	22.1	130	5.5	1,146	48.8
Widower	51	2.2	74	3.1	9	0.4	134	5.7
Divorced	50	2.1	67	2.9	22	0.9	139	5.9
Total	912	38.8	1,131	48.1	307	13.1	2,350	100

The traffic noise perception found that 48.1% of the participants responded that they do perceive it. The answer NO was identified in 38.8% of the participants, while those who responded that they Do Not Know resulted in 13.1%. The last two values stand out as a whole, which reveals that slightly more than half of the participants, 51.9%, do NOT identify or perceive the presence of noise, despite living in areas exposed to values higher than 70 dBA.

The demographic data allow us to identify the characteristics of the population that perceives traffic noise. Due to gender, 28.8% of women and 19.3% of men responded that they perceive traffic noise, representing 48.1% of the participants.

In the different age groups, 18–30 category (16%) of the participants responded that they do NOT perceive traffic noise, while in the other categories, 31–45 (12.8%); 46–60 (13.9%); >60 (6.1%), the highest response was YES. Such results demonstrate that the younger population has a lower appreciation of traffic noise.

In the schooling of the participants, the categories High School (8.9%) and University (7.3%) stand out, in which the participants responded that they do not perceive traffic noise. In the other educational levels, namely, Uneducated (9.1%); Elementary (11.1%); Junior High School (10.8%); and Post-graduate (0.5%), the participants responded that they do perceive the noise. Therefore, the results show that for the participants at a medium–high educational level, noise does not represent a factor that causes any type of displeasure or annoyance.

According to the occupation categories of the participants, namely, Unemployed (6.5%); Worker (22.3%); Retired (3.6%); Housewife (12.0%); and Student (3.7%), the participants responded that they do perceive traffic noise. Only in the Strike category (0.2%), they responded that they do not perceive the noise. This evidence that the different occupation categories of the participants, especially those employed and housewives are more accustomed to noise perception due to their daily activities.

In all marital status categories, namely, Married (22.1%); Single (20.0%); Widower (3.1%); and Divorced (0.9%), the participants responded that they do perceive noise. In this way, it implies that marital status may be related to some occupation, and consequently they have a better appreciation of the noise around them.

To describe the relationship between the variables, the Pearson's correlation test was used, finding that the noise levels and traffic noise perception represent very low correlations but with significant values of <0.05. The detailed results are discussed in Table 3.

**Table 3:** Correlations.

	**Level noise**	**Noise perception**	**C.1**	**C.2**	**C.3**	**C.4**	**C.5**	**C.6**
Level noise	1							
Noise perception	0.080^**^	1						
C.1 Considering outside noise	0.045^*^	0.519^**^	1					
C.2 Considering indoor noise	0.150^**^	0.384^**^	0.318^**^	1				
C.3 What times do you find the noise most annoying?	0.080^**^	1.000^**^	0.519^**^	0.384^**^	1			
C.4 Do you consider that your street, compared to previous years, is on average…	0.012	0.263^**^	0.405^**^	0.148^**^	0.263^**^	1		
C.5 Do you consider that your street, compared to the rest of the city, is on average…	−0.039	0.299^**^	0.281^**^	0.329^**^	0.299^**^	0.200^**^	1	
C.6 The street noise annoys or disturbs you the most when you are…	−0.103^**^	−0.180^**^	−0.113^**^	−0.167^**^	−0.180^**^	−0.134^**^	−0.082^**^	1

*Pearson correlation*.

** *Correlation is significant at the 0.01 level (2-tailed)*.

* *Correlation is significant at the 0.05 level (2-tailed)*.

The noise levels evaluated exceed 70 dBA in contrast to the perception of noise of the participants denotes a very low correlation, despite being significant. This implies that people do not pay attention and are even accustomed to the problems they are exposed to daily derived from vehicular traffic.

The variables “considering exterior noise” and “interior noise” also presented low, although significant correlations. Hence, this may imply that people confuse noise coming from different sources, impacting their perception of noise.

The variable concerning the time of the day they find noise most annoying represents a very low significant correlation with noise levels. However, a perfect positive correlation was found with noise perception.

Considering their street compared with the previous years showed no significant correlation concerning noise levels but did show a low significant correlation in terms of perception. Similarly, is the consideration of their street compared with the rest of the city? Therefore, people neither have a clear idea on the effect of traffic noise in previous years, nor do they have an evident appreciation of the noise of their street compared with other places in the city.

The annoyance or disturbance of street noise when inside or outside the home presented a significant negative correlation concerning noise levels and noise perception; this means that the participants cannot distinguish exposure to traffic noise, regardless of their location at home, and the perception of the problem.

## Discussion

The vehicular traffic noise is an essential factor in noise annoyance perception (Dzhambov and Dimitrova, 2018). When the perception of noise annoyance is very high, traffic noise, especially from heavy vehicles or ambulance sirens, is easily identified as annoying (Cramer et al., 2019). Sensitivity to traffic noise annoyance is higher in those exposed to noisier streets (Kishikawa et al., 2006). In the study, more than half of the participants do NOT perceive or identify noise as an annoying factor. This conclusion is essential because the participating population lives in areas close to high-traffic roads. The traffic noise levels evaluated throughout the study exceeded 70 dBA. Such assumption suggests that the population does not consider traffic noise as a problem they should be affected. A potential explanation could be that people are desensitized to the adverse conditions of the traffic noise due to the impossibility of changing their environment or changing their residence.

The first consideration in the perception of annoyance should be the age of the participants, i.e., for those who are younger have less problem with the sounds, while those who are older, traffic noise does represent an annoyance (Dzhambov et al., 2017). Our results identify that those under the age of 30 years do not perceive traffic noise, while the rest recognize it. Consequently, it reflects that age is an aspect that affects the comprehension. As age increases, the perception of traffic noise increases. It is common for younger people to frequent leisure places that are noisier. Therefore, they become desensitized to noise from any source, which does not recognize it as something annoying or harmful to their health, but these assumptions will have to be evaluated in a subsequent study.

That is why the urban centers are the ones with the highest traffic noise, where levels of 55 dBA are exceeded by far during the day and 45 dBA at night (Kim et al., 2012). Some studies conclude that in areas exposed to vehicular traffic, noise can exceed 73.1 dBA, while in quiet or low-traffic areas, the noise levels oscillate around 64 dBA (Paiva et al., 2019). The outcomes of this research address the traffic noise generated during the day, and it can be seen in Table 1 that the different areas evaluated have the values above 70 dBA. It can also be noted that the highest noise values include the most increased traffic of heavy vehicles.

The research reflects the opinion of those participants who do identify it, but at the same time, consider that the street where they live is less noisy than the rest of the city, which would imply that the participants do not have a clear idea of the intensity of traffic noise. This statement coincides with other works where they describe that people who demonstrate high annoyance from traffic noise are those living in areas with noise above 65 dBA (Di et al., 2012). Even the perception of annoyance does not change if a range of 45–95 dBA is considered. However, categories could be established in the intensity of annoyance, such as minor annoying, annoying, and very annoying, depending on the intensity of the noise (Camusso and Pronello, 2016). Likewise, noise levels found in dense traffic areas vary within the range of 80–85 dBA, while interviews found that 48.4% of the population expressed a high degree of noise annoyance (Paiva et al., 2019).

The sensitivity to traffic noise is greater during the night periods, as it causes an interruption of sleep; however, the annoyance by traffic noise is perceived during the day, when the inhabitants are inside their homes, in rooms, or in rest areas close to the street (Jakovljevic et al., 2009). Thus, the inhabitants cannot be at ease inside their homes (Camusso and Pronello, 2016). Some proposed models describe that inhabitants consider traffic noise above 70 dB as a highly annoying problem outside their homes. When the traffic noise level is higher than 76 dB, inhabitants consider the noise to be highly annoying when they are indoors (Fyhri and Klæboe, 2006). The previously described factors coincide with the present results. It can be identified that people recognize that the annoyance noise is more significant when being in the interior of their house during the night periods. Probably, it is because the traffic noise interferes with the tranquility of the people when they are inside their homes to dedicate part of their time to rest.

## Limitations

This research was carried out with developed activities during the day periods, which leaves night periods that allow the development of complete 24-h period calculations unobserved.

It is also required to increase some variables related to the infrastructure of the homes, such as construction materials, isolation, number of windows, bedrooms position, and living rooms concerning the street or noise source.

The results are focused on the noise generated by vehicles in transit, but those areas where high sounds are generated outside their facilities or use sound amplifiers were not considered. Besides, improvements should be made in the description of vehicle flow, such as speed and duration at traffic lights.

## Conclusion

The vehicle type and flow are the essential elements for the generation and increase of traffic noise levels. The study identifies that more than half of the participants from Matamoros city do NOT perceive traffic noise as a factor that causes them annoyance.

Women show a better appreciation of traffic noise perception, while younger people show a higher tolerance for high-level exposure. The participants do not differentiate between the different types of noise, nor do they recall the noise situation from previous years, making it difficult to determine a pattern for comparison. Thus, it may reflect the evident lack of necessary information among the vulnerable population about the noise problem and the probable consequences for health in the short, medium, and long terms.

Due to the questioning performed and the results of the very low correlations, the simple opinion of the perception of traffic noise is not sufficient to make predictions or generalizations.

The development of ongoing socioenvironmental studies related to traffic noise must have advantages in different aspects. First, it allows describing the vehicular flow in certain areas, which allows the development of better planning of the road traffic. Second, it allows knowing the parameters of environmental noise in their different schedules, which allows the local authorities to have references that facilitate the development of regulations and, in their case, the verification of the established limits.

In contrast to what is described in the literature, people in Matamoros do not care about noise pollution, so more multidisciplinary research should be conducted to comprehend the possible reasons behind that issue, allowing novel noise mitigation strategies to be developed. These projects must have the active participation of the authority in urban planning, transportation, and the environment to prevent the future complaints of the upcoming residents, especially in vulnerable areas.

Finally, the voluntary participation of the citizens in the development of this type of project is required since their collaboration is vital, representing a challenge for researchers to find strategies that allow them to expand the sample in subgroups, new variables, and research lines.

## Data Availability Statement

The original contributions presented in the study are included in the article/Supplementary Material, further inquiries can be directed to the corresponding author/s.

## Ethics Statement

The studies involving human participants were reviewed and approved by Research and Ethics Committee of the Unidad Académica Multidisciplinaria Matamoros of Universidad Autónoma de Tamaulipas. The patients/participants provided their written informed consent to participate in this study.

## Author Contributions

BZ-G and FP-C carried out the study design, data analysis, and writing of the manuscript. VP-S and YV-N developed the statistical analysis, participated in the writing, and editing of the manuscript. JV-M designed the database, supervised the correct collection, transcription of data, and reviewed and corrected the manuscript. LR-R and OM-A participated in the supervision of the fieldwork and the correction of the manuscript. All the authors approved the final version of the manuscript. All the authors were involved in different stages of the study.

## Conflict of Interest

The authors declare that the research was conducted in the absence of any commercial or financial relationships that could be construed as a potential conflict of interest.
