# Supplementary material for: Traffic Noise Annoyance in the Population of North Mexico: Case Study on the Daytime Period in the City of Matamoros
Source: Front Psychol. 2021 May 24;12:657428. doi: 10.3389/fpsyg.2021.657428 (PMC8180601; doi:10.3389/fpsyg.2021.657428)
Supplement: Supplementary file 1 [file Data_Sheet_1.docx]

UNIVERSIDAD AUTÓNOMA DE TAMAULIPAS

UNIDAD ACADÉMICA MULTIDISCIPLINARIA MATAMOROS UAT

“RUIDO URBANO Y SUS EFECTOS EN LA POBLACIÓN”.

Estimado participante:

La presente encuesta tiene la finalidad de obtener información sobre: Ruido urbano y sus efectos en la población de Matamoros, Tamaulipas. Su participación es muy valiosa, por lo que le pedimos la mayor honestidad al responder. La información que resulte será manejada de manera anónima y confidencial. Agradecemos su participación.

RED PRECONRI

Prevención de Conductas de Riesgo

Tiempo estimado para responder: 5 minutos.

**Control.**

| A.1 No de cuestionario: |  |  | A.2 Fecha: | - - |  | A.3 Código de encuestador: |  |
| --- | --- | --- | --- | --- | --- | --- | --- |

**Ubicación.**

| B.1 Domicilio: |  | | |
| --- | --- | --- | --- |
| B.2 entre: |  | B.3 y |  |
| B.4 colonia: |  | | |

**Datos del encuestado.**

| Edad: |  |  | Género: | Masculino | Femenino |
| --- | --- | --- | --- | --- | --- |

| Estado civil: | Soltero | Casado | Viudo | Divorciado |
| --- | --- | --- | --- | --- |

| Nivel de estudios: | Sin estudios | Primaria | Secundaria | Bachillerato | Universidad | Posgrado |
| --- | --- | --- | --- | --- | --- | --- |

| Situación laboral: | No trabaja | Trabaja | En paro | Jubilado | Ama de casa | Estudiante |
| --- | --- | --- | --- | --- | --- | --- |

**Percepción del ruido.**

| C.1¿Considera o percibe que el ruido de tráfico es molesto? | No | Sí | No lo sé |
| --- | --- | --- | --- |
| C.2 Considerando el ruido exterior, ¿Cuál perioro le resulta más molesto el ruido? | Diurno | Nocturno | 24 horas |
| C.3 Considerando el ruido interior, ¿Cuál periodo le resulta más molesto el ruido? | Diurno | Nocturno | 24 horas |
| C.4 De manera general, ¿Qué horario le resulta más molesto el ruido? | Diurno | Nocturno | 24 horas |
| C.5 Considera que su calle, respecto al resto de la ciudad, es en promedio… | Menos ruidosa | Igual de ruidosa. | Más ruidosa. |
| C.6 Considera que su calle, respecto a años anteriores, es en promedio… | Menos ruidosa | Igual de ruidosa. | Más ruidosa. |
| C.7 El ruido de la calle le molesta o perturba cuando usted está… | Dentro de su casa |  | Fuera de su casa |

UNIVERSIDAD AUTÓNOMA DE TAMAULIPAS

UNIDAD ACADÉMICA MULTIDISCIPLINARIA MATAMOROS UAT

“URBAN NOISE AND ITS EFFECTS ON THE POPULATION”.

Dear Participant:

The present survey has the purpose of obtaining data on: Urban noise and its effects on the population of Matamoros, Tamaulipas. Your participation is very valuable, so we ask you to be as honest as possible. The resulting information will be handled anonymously and confidentially. We appreciate your participation.

PRECONRI research group

Risk Behavior Prevention

Estimated time to respond: 5 minutes.

**Control.**

| A.1 Survey #: |  |  | A.2 Date: | - - |  | A.3 Surveyor code: |  |
| --- | --- | --- | --- | --- | --- | --- | --- |

**Ubicación.**

| B.1 Address: |  | | |
| --- | --- | --- | --- |
| B.2 between: |  | B.3 & |  |
| B.4 Section: |  | | |

**Datos del encuestado.**

| Age: |  |  | Gender: | Male | Female |
| --- | --- | --- | --- | --- | --- |

| Marital status: | Single | Married | Widower | Divorced |
| --- | --- | --- | --- | --- |

| Education level: | Uneducated | Elementary | Junior High School | High School | University | Postgraduated |
| --- | --- | --- | --- | --- | --- | --- |

| Employment status: | Unemployed | Worker | Strike | Retired | Housewife | Student |
| --- | --- | --- | --- | --- | --- | --- |

**Percepción del ruido.**

| C.1¿ Do you consider or perceive traffic noise to be annoying? | No | Yes | I do not |
| --- | --- | --- | --- |
| C.2 Considering outside noise, which period do you perceive the noise most annoying? | Daytime | Night-time | 24 hours |
| C.3 Considering indoor noise, which period do you perceive the noise most annoying? | Daytime | Night-time | 24 hours |
| C.4 Commonly, what times do you perceive the noise most annoying? | Daytime | Night-time | 24 hours |
| C.5 Considers that its street, with respect to the rest of the city, is on average … | Less noisy | Equally | Noisier |
| C.6 Do you consider that your street, compared to previous years, is on average | Less noisy | Equally | Noisier |
| C.7 The street noise annoys or disturbs you the most when you are.. | Inside your home |  | Outside your home |
